# Book Reviews

**Published:** 1885-09

**Authors:** 


### BOOK REVIEWS.

ON A NEW METHOD OF RECORDING THE MOTIONS OF THE SOFT PALATE. *By* HARRISON ALLEN, M. D., *Professor of Physiology in the University of Pennsylvania. Extracted from the Transactions of the College of Physicians of Philadelphia. Third series, volume vii. Octavo, pp. 34. Philadelphia :* P. BLAKISTON, SON & CO. 1884.

Within the few pages of this monograph are comprised the results of very many hours of patient toil and highly original research. A straight rod, eight inches in length, is passed through the nose, from before backward, until its bulbous extremity reaches into the naso-pharynx. The end which remains without the nostril is then elevated so that the rod is brought in contact with the anterior nasal border, and it is there retained by a wire attachment to a head-band, such as is ordinarily employed for the support of laryngeal head-reflector. With the rod in this position, each elevation of the palate which is caused during phonation and by the acts of exhaling, coughing, deglutition, etc., produces a corresponding depression of the external end of the rod, and *vice versa.* The free end of the lever is brought in contact with the carbon covered cylinder of the Ludwig kymographion, and the cylinder is made to revolve by means of the clock-work of the apparatus, when there appears a distinct tracing, similar to the markings of a sphygmograph.

Just as the sphygmographic tracing indicates the character of the pulse wave, so the tracing of the Palate-Myograph, as the new apparatus is designated, correlates with the varied movements of the soft palate in the articulation of the different sounds of vowels, different letters, words, and phrases.

It is suggested that the palate-myograph may prove useful in studying the mechanism of the soft-plate in disease as well as in health: in determining the degree of degeneration of the levator palate muscles in progressive dry aural catarrh, for the study of stammering, and for the detection of paralysis of the palate. It is also more than hinted that the method may be made available for the comparative study of language, for the instruction of the deaf, and for the formation of a system of logography. W. E. Casselberry.

---

A Hand-book of Pathological Anatomy and Histology. *By* Francis Delafield, m. d., and T. Mitchell Prudden, m. d. *Published by* Wm. Wood & Co., *New York.*

The authors in their preface claim the present work to be a second edition of what has been known as "Post Mortem Examinations and Morbid Anatomy, by Francis Delafield." So much new material has been added that it may properly be regarded as a new work rather than a new edition of an old one.

The subjects discussed by the authors are comprised in four parts. The first is devoted to a description of the method of making post mortem examination, both of the adult and of new-born infant, as well as of cases of suspected poisoning. The methods of preserving and preparing tissues for future study is also given here.

The second part embraces the subjects grouped by other authors commonly under the name of *general pathology.* In this part there is very much that is new, or not to be found in the first edition.

The third division embraces the morbid anatomy of the organs, and the fourth, "lesions found in the general diseases; in poisoning and in violent deaths."

Much must be said in praise of this work. It describes briefly, clearly and pleasantly a great variety of lesions. It is unusually well illustrated. The cuts have been made by processes of photo-engraving, from drawings, by the authors themselves, from actual specimens. They are particularly good as drawings of microscopical objects.

A single volume covering so extensive a subject as this can not be so exhaustive as the more extensive and complete works, such as we already have. It will, however, be found both by students and practitioners an excellent hand-book.

The subject of inflammation is not treated of as we ordinarily find it in other works on pathology, and we think the new method is not commendable. No description is given of typical inflammation, but eight varieties are described separately. A student beginning the study of pathology with this text-book would probably wonder why these eight different lesions were called inflammations and what there was in common to them all. They are all *products* of inflammation. In order to make this manifest it would have been better to have pointed out distinctly the factors common to all, and then to have made plain the differences which constitute these varieties.

The appearance of the latter portion of the book, especially, is marred by the amount of blank paper that is left at the top and bottom of some pages. For instance, gout is discussed in nine lines, which is all the printing that is placed on one page. The book might have been much smaller, without sacrificing the subject-matter, if such blank spaces had been omitted.

N. S. D., JR.

---

MINOR SURGICAL GYNECOLOGY. A TREATISE OF UTERINE DIAGNOSIS, AND THE LESSER TECHANICALITIES OF GYNECOLOGICAL PRACTICE, INCLUDING GENERAL RULES FOR GYNECOLOGICAL OPERATIONS AND THE OPERATIONS FOR LACERATED CERVIX AND PERINEUM, AND PROLAPSUS OF UTERUS AND VAGINA. *By* PAUL F. MUNDÉ, M. D. *Octavo, pp. xxii, 552. New York:* WILLIAM WOOD & CO. 1885. *Chicago:* W. T. KEENER.

The second edition of Dr. Mundé's MINOR SURGICAL GYNECOLOGY supplies a want felt for a long time by every practitioner who has given attention to the diseases of women. The details of gynecological technique and practice are faithfully described, in terms which every one can comprehend. The labor involved in the preparation of such a book is simply enormous. Dr. Mundé has placed the profession under lasting obligation for the production of a book which has no peer in German, French or English.

The books is not without faults; omissions are numerous. BANDL'S methods of treatment of uterine inflammations deserve consideration. DR. D. BERRY HART makes the following assertion in his "Atlas of Female Pelvic Anatomy" with reference to the genu-pectoral posture: "There is no assertion more often made than that the retroverted unfixed uterus becomes replaced, *i. e.*, anteverted, when the genu-pectoral pasture is assumed and the vaginal orifice opened up. This is an undoubted error. The uterus really moves further from the pelvic outlet, and becomes more retroverted." While Dr. HART'S statement may be taken *cum grano salis*, it deserves attention.

In the selection of terms, and structure of sentences, there is room for criticism. Finally, the nominative case of the pronoun of the first person recurs with a frequency at once unpleasant and in bad taste. W. W. J.

---

A TREATISE ON ABDOMINAL PALPATION, AS APPLIED TO OBSTETRICS, AND VERSION BY EXTERNAL MANIPULATIONS. *By* A. PINARD, M. D., *Associate Professor in the Faculty of Medicine of Paris, etc. Paris, 1878. Translated by* L. E. NEALE, M. D., *Chef of the Obstetrical Clinic and Demonstrator of Obstetrics in the University of Maryland. 12mo, pp. 101. New York:* J. H. VAIL & CO. 1885.

The raison d'etre for Dr. NEALE'S translation of PINARD'S lectures on Abdominal Palpation is obvious. We have many excellent English text-books on obstetrics, but no adequate exposition of abdominal palpation as practiced in Austria, France and Germany. The papers of MUNDÉ, RICHARDSON, WRIGHT, CHADWICK and WILSON, while meritorious, have not supplied the hiatus.

We venture to predict for the brochure before us, great practical utility to the profession, and extensive adoption as a text-book. W. W. J.

